# Starting a spine surgery service in a low-resource setting: a look back over twenty five years of spine surgery in Malawi

**DOI:** 10.1007/s00264-022-05339-8

**Published:** 2022-03-01

**Authors:** Chris Lavy, Nyengo Mkandawire

**Affiliations:** 1 Nuffield Department of Orthopaedics Rheumatology and Musculoskeletal Science, Oxford University, Oxford, UK; 2 Malawi College of Medicine, Blantyre, Malawi

**Keywords:** Spine surgery, Low- and middle-income country (LMIC), Low-resource setting

## Abstract

**Purpose:**

This is a short historical narrative of the development of spine surgery in Malawi.

**Methods:**

The authors communicated and both drew on memories and anecdotes over the last 25 years.

**Results:**

This is not a scientific paper, so there are no results.

**Conclusion:**

The short paper outlines the development of spine surgery in Malawi over the last 25 years. This develops in association with the overall increase in the number of surgeons in the country.

## Introduction

The development of a spine surgery service in a low-resource setting has to be seen in the context of the country’s health services as a whole. Twenty-five years ago in 1996, Malawi had a population of around 12 million, one orthopaedic surgeon and no practising neurosurgeons. The country’s only medical school started in 1990 and was only just starting to produce doctors. In such a situation, however keen on spine surgery the one orthopaedic surgeon was, he or she had to consider the competing health needs of the population as well as other orthopaedic service needs. Over the years, more orthopaedic surgeons have been trained, and at the time of writing, Malawi has 15 orthopaedic surgeons, four neurosurgeons, six orthopaedic surgeons in training, and six neurosurgeons in training for its 18 million people. This account is not in any way a ‘how to do it’ manual, but it is a personal account of starting a spine surgery service in a situation where it was clearly needed, but resources were limited. With hindsight, maybe mistakes were made, and we will be the first to admit that, but this article charts the first steps in what is a service that has grown a lot, and we hope will continue to grow, and serve the people who need it.

## Priority setting

We have discussed above the competing demands on a small number of orthopaedic surgeons. Limb trauma, especially that involving economically active young adults who are also responsible parents, is always a major priority for theatre time and space, as is paediatric orthopaedic surgery that gets children onto their feet and into school. Elective spine surgery is a low priority. Emergency spine conditions such as acute trauma, TB and other infections, and other treatable spinal conditions that cause rapid neurological deterioration press for the development of a spine service. With limited surgical resources for an adequate spine service, the development of a spine rehabilitation service is of equal importance in order to maximise the outcome of both operated and non-operated patients.

## Manpower

**Fig. 1:**
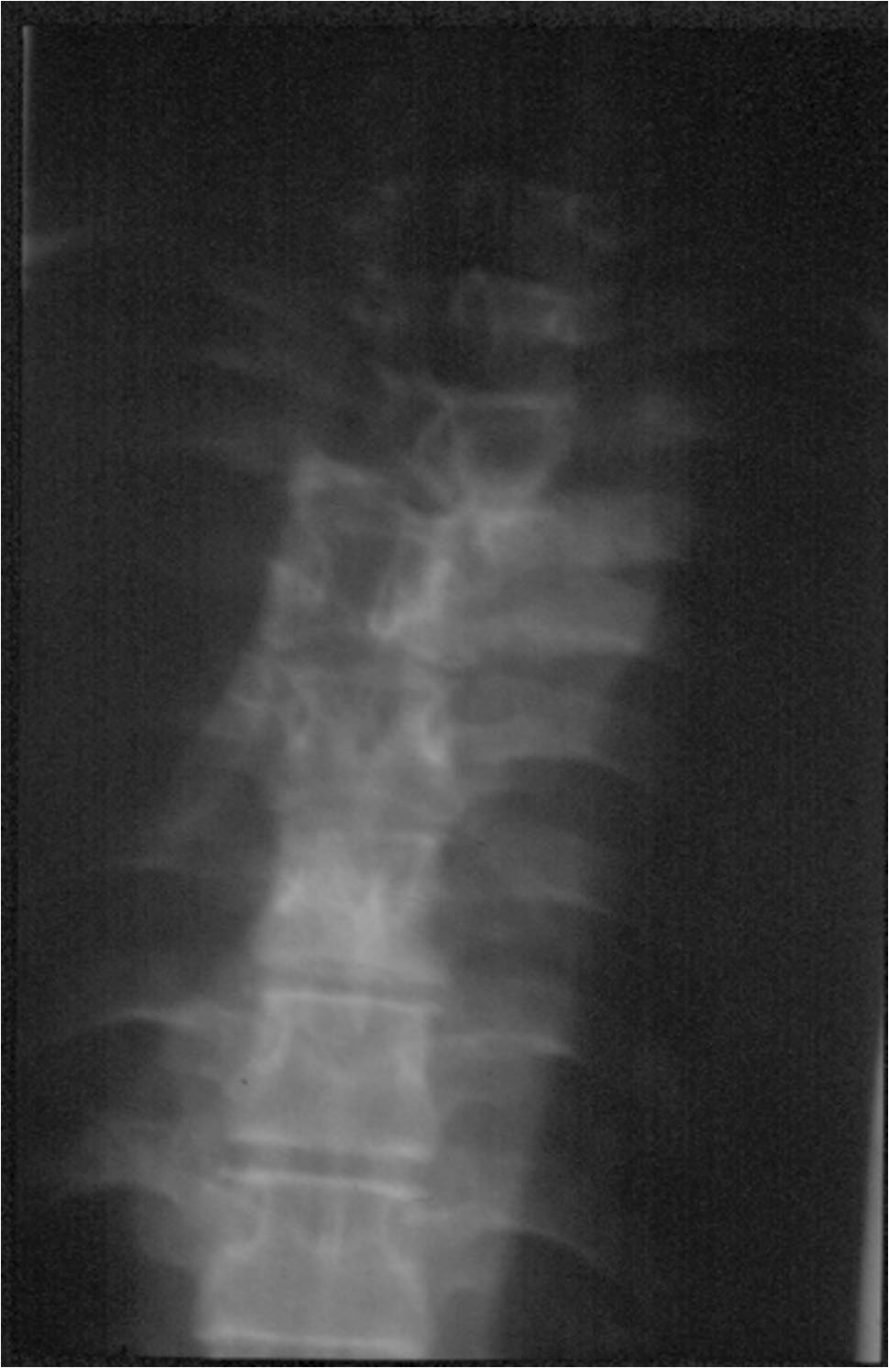
A case of TB of the thoracic spine where there was a lateral shift of the midthoracic spine and complete paraplegia with incontinence. Posterior decompression and in situ fusion with bone graft resulted in complete recovery of function

**Fig. 2:**
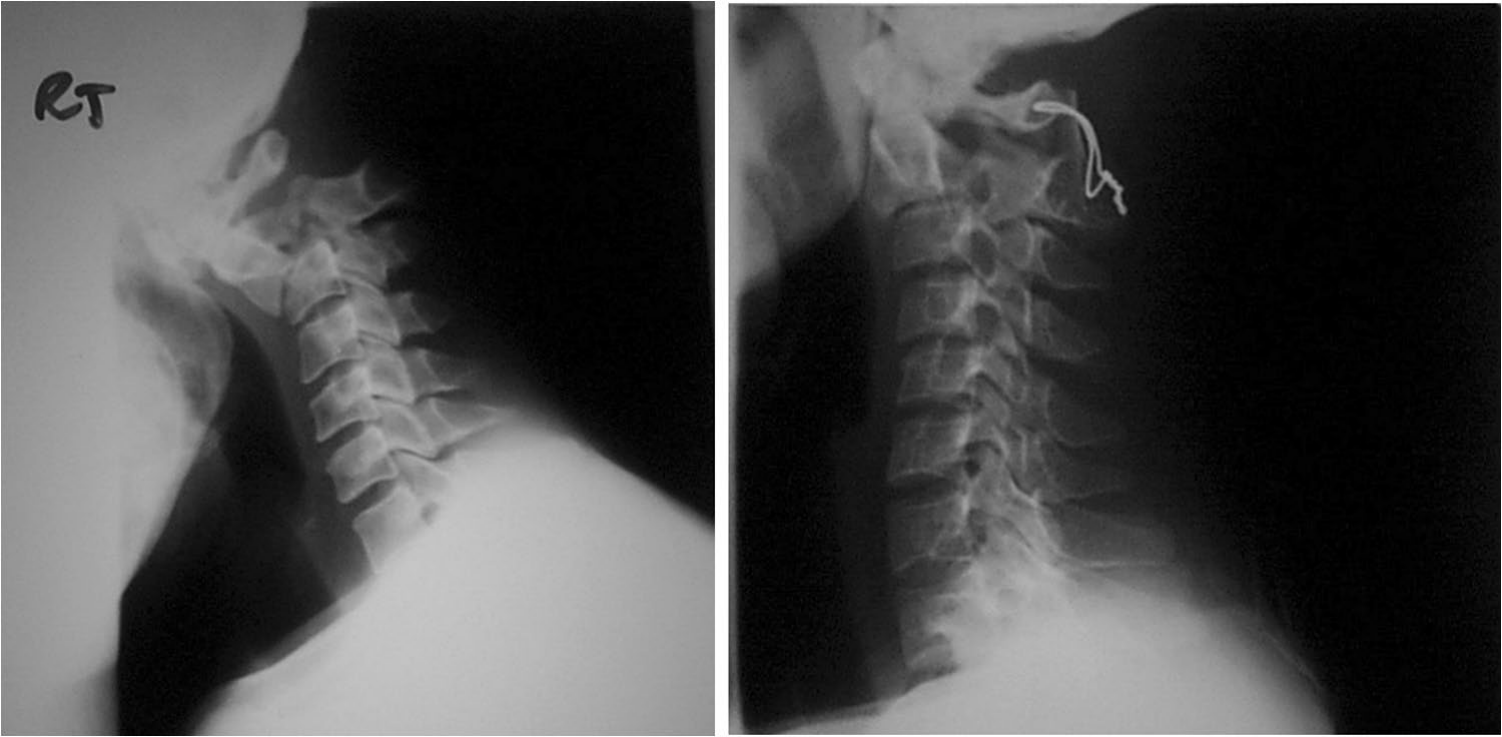
A C1 to C2 fusion for unstable C2 fracture fixed with stainless steel wire and bone graft

**Fig. 3:**
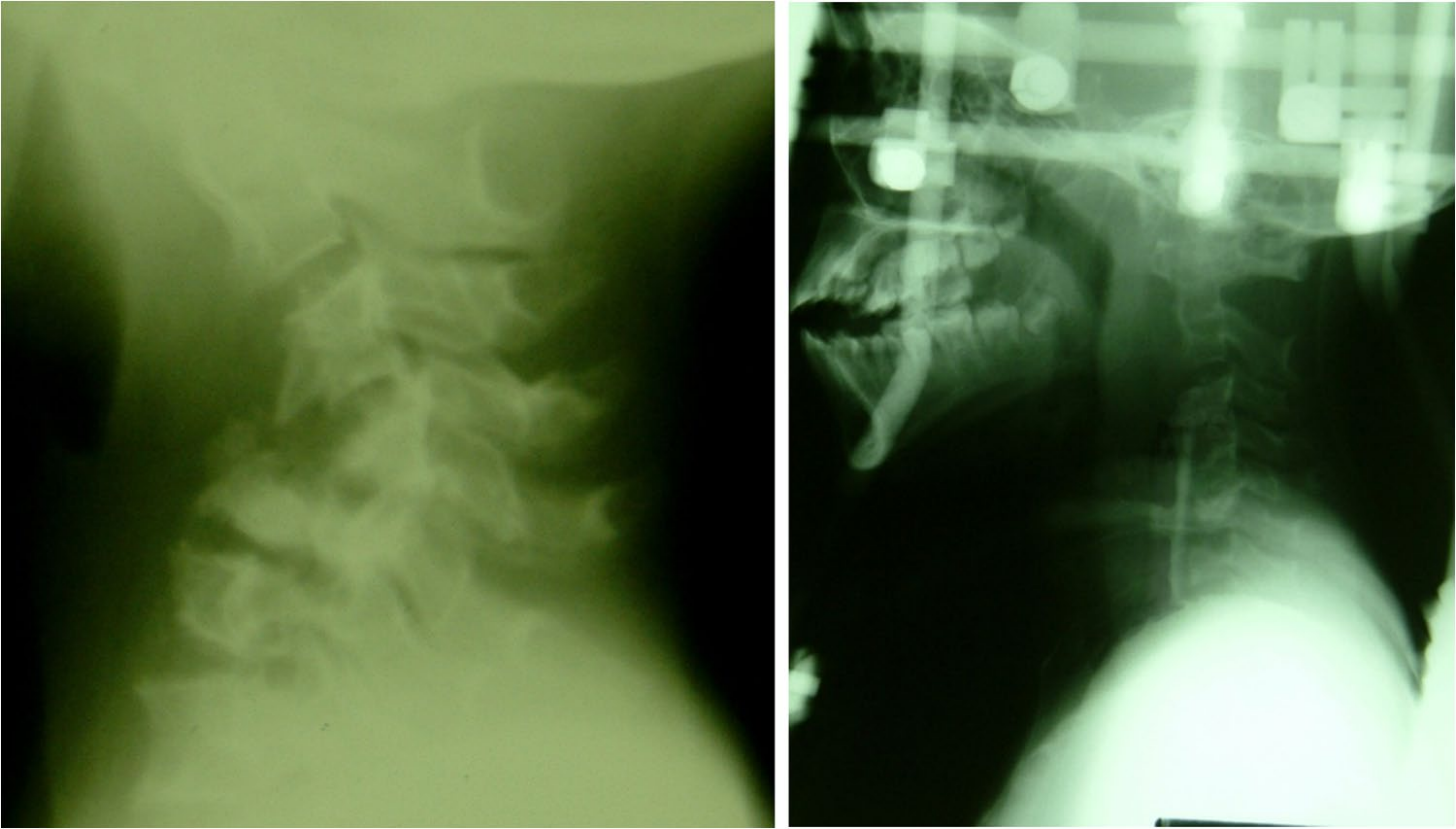
A case of cervical spine TB with kyphosis and spinal cord compression treated by anterior decompression, fibular graft, and donated halo jacket stabilisation. Immediately post surgery, her lower limb function recovered

**Fig. 4:**
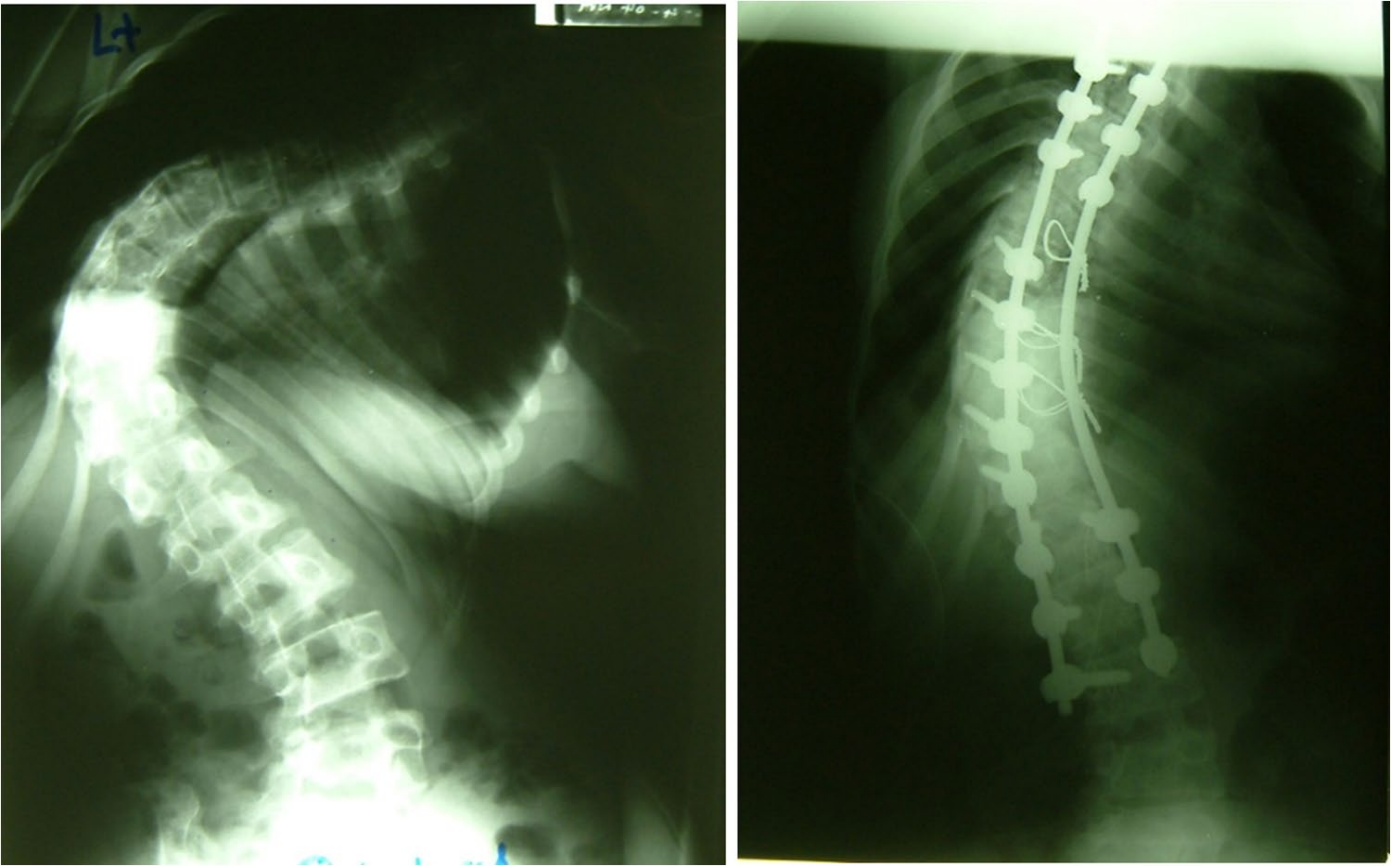
A case of severe scoliosis correction resulting in a significant improvement albeit not perfection

To complement the services of the limited number of orthopaedic surgeons, Malawi trains a cadre of nonphysician clinicians called orthopaedic clinical officers or OCOs [1]. These clinicians are the first port of call for most orthopaedic conditions for 85% of the Malawi population, which is rural based, at the district hospital level. They are trained in appropriate surgical techniques for orthopaedic emergencies such as debridement of an open fracture or reduction of a major joint dislocation. The emphasis in their training is to provide conservative nonoperative orthopaedic care in the rural areas and to refer cases that need surgical care to orthopaedic surgeons who are primarily based at the tertiary referral centre. Whilst they would not approach the spine surgically, OCOs are taught to treat spinal injuries conservatively, and many patients with unstable fractures but no neurological deficit have returned to manual labour after bed rest and appropriate immobilisation or bracing until the fracture is united. Just as most orthopaedic cases in the district hospital setting are treated by OCOs, the majority of anaesthesia care is performed by similar nonphysician anaesthesia clinicians called anaesthetic clinical officers or ACOs [2]. With appropriate training, mentorship and supervision, ACOs can provide safe anaesthesia as illustrated in the cases outlined. The cases illustrated in Figs. 1, 2, 3, and 4 were managed by anaesthetic clinical officers.

## Imaging

Twenty-five years ago, the only spinal imaging was plain X-rays. For conditions such as TB spine or spinal abscesses where there is a neurological deficit, X-rays are clearly not as accurate as more modern imaging techniques but can show soft tissue shadows in the region of the abscess and can guide a surgical approach to the right level where it is indicated. This is not the place to go into detail over management of conditions such as TB, but even in cases with severe neurological deficit, the initial management is always to start antibiotic treatment, and many cases improve. As time went by, imaging facilities improved and a C-arm image intensifier became available in the teaching hospital. A CT scan and later an MRI scan was also purchased, although the maintenance of these complex machines is difficult, and they are not always available.

For patients with financial ability, spinal treatment abroad was always available. For the not so rich, who could not afford full treatment abroad but who could afford a few hundred dollars for a day trip to Johannesburg and an MRI scan, this was an option to at least get a diagnosis and imaging. The authors have operated on many cases of discectomy and spinal stenosis with this hybrid system. The advantage is that the patient gets state of the art imaging and diagnosis, then a surgical procedure that they can afford, in their own country where they can be supported by family and friends.

In the early days with no image intensifier, even per operative level checking was difficult, but we managed with plain X-rays on a portable machine.

## Techniques, equipment, and instrumentation

With limited resources, it is important to minimise risk, and we found that the costotransverse approach was the most patient-friendly way to approach vertebral body infections. With care, the pleural cavity can be spared, vertebral body infection cleared, and graft can be placed anteriorly followed by immobilisation or bracing.

We did the early procedures using donated retractors, curettes, and spinal rongeurs. NHS hospitals upgrading their spinal sets were a very fertile source of instruments. As we were starting to use pedicle screws, the Western world was moving away from stainless steel to titanium, and we were donated a number of old pedicle screw sets that were still very serviceable.

## Training

Both authors are grateful to many visiting surgeons particularly from the USA and the UK who came usually for a month at a time and were happy to operate with us. Our role was to keep them attuned to the local situation; for example, before the establishment of a transfusion service ten years ago, blood transfusion had to be arranged ahead of time with patients’ relatives as donors. The visitors played an important role teaching us new techniques. We also learned from visits to regional hubs such as Cape Town and Durban.

As we became more comfortable handling and approaching the spine, we progressed under supervision to tackling deformity. There was no spinal cord monitoring so we used the wake-up test after instrumentation and correction. This involved letting the anaesthesia get light enough after reasonable correction to ask the patient to move their feet, and if they could, we accepted the position. The aim of scoliosis correction was to improve the patient’s overall deformity and not to go for perfection with the risks to the spinal cord that that involved.

Training is not just learning for oneself but also passing it on. As more doctors are trained in the country, the number interested in orthopaedic surgery increases, and the opportunity to train and mentor such trainees increases. We have also been fortunate to make links for training with other countries, and two locally trained orthopaedic surgeons have recently been sent for spine fellowships at centres in India and Egypt, respectively.

## The future

The exciting world of spinal orthopaedics is starting to flower in Malawi. As it does, and as imaging, instrumentation, and theatre conditions improve, the nature of the work starts to resemble spinal orthopaedics in more affluent countries. At present, a large part of the surgical work continues to be TB spine, and this has been for both authors the most rewarding part of spinal practice, as the combination of anti-TB therapy and decompression/stabilisation brings recovery of function in the majority of cases. Other cases now being routinely treated include epidural abscess, extramedullary spinal cord lesions, disc herniations, cauda equina syndrome, spinal stenosis, and many other degenerative conditions.

Neurosurgery has lagged behind orthopaedic surgery in Malawi but is now starting to develop, and there is now a local neurosurgical training programme supervised by 4 neurosurgeons trained regionally in South Africa (1), Ethiopia/Tanzania (1), and Morocco (2). Currently, there are 6 neurosurgeons in training on the local programme. In many developed countries, the spine is the site of ‘turf wars’ between orthopaedics and neurosurgery. Given the shortage of supply in both disciplines and the friendly cooperation between colleagues, we hope to develop the service together and to benefit the people who so badly need it. An exciting development is the soon-to-be-opened Lilongwe Institute of Orthopaedics and Neurosurgery, which will have state-of-the-art orthopaedic and neurosurgery services and will be the major hub for training in these specialities. The facility will open in 2022.

## Data Availability

There is no data store for this paper.
